# The Relation of the Dentist to the Nose, Throat, and Ear Specialist2An address delivered before the Harvard Odontological Society, December 15, 1904.

**Published:** 1905-02

**Authors:** George L. Richards

**Affiliations:** Fall River, Mass.


### International Dental Journal.

VOL. XXVI. FEBRUARY, 1905. NO. 2.

### Original Communications.[1]

### THE RELATION OF THE DENTIST TO THE NOSE, THROAT, AND EAR SPECIALIST.[2]

BY GEORGE L. RICHARDS, M.D., FALL RIVER, MASS.[3]

GENTLEMEN,—I have long been of the opinion that the separation of dentistry as a branch of medicine from general medicine and general surgery is a mistake. I believe that dentistry should be considered only as one of the departments of general medicine and surgery in the same manner that the practice of the diseases of the ear, nose, and throat is so considered. I believe that the time will come when the dentist will first be graduated in the general medical course, or at least carried farther in it than he is at present. The Harvard Dental School requires much more than the average, in that the first year in the dental school and the first

[1] The editor and publishers are not responsible for the views of authors of papers published in this department, nor for any claim to novelty, or otherwise, that may be made by them. No papers will be received for this department that have appeared in any other journal published in the country.

[2] An address delivered before the Harvard Odontological Society, December 15, 1904.

[3] Otologist and Laryngologist to the Fall River Union Hospital; Fellow American Otological Society, American Laryngological, Rhinological, and Otological Society, etc.

year in the medical school are one and the same as to course. I think the time will come when the institution will go still farther and that the rudiments at least of general medicine and surgery will be taught to the students of the dental school.

I have long believed that it would be better for our patients if we who are workers in the upper air-tract, the so-called nose, throat, and ear specialists, had a better knowledge of the teeth and their influence relative to these affections, and I think it is equally true that the dentist ought to know more of the affections of the nose, throat, and ear than he does at present.

I therefore welcome this opportunity to speak to you in regard to some of the affections in the treatment of which it seems to me we meet or ought to meet on a common ground.

### AURAL NEURALGIA OF DENTAL ORIGIN.

It is well known that the fifth or trifacial nerve has very intimate relationships with all the rest of the face through its various ganglia, its own complex mechanism, the extent of its distribution, and its relations with the sympathetic. Many cases of facial neuralgia have their entire origin in the teeth, even though the patient makes no complaint whatever of the teeth.

I have also found that quite a number of ear-pains have their point of origin in the teeth, although I have been able to find but little touching this subject in the various treatises on diseases of the ear. In my own clinical experience it has seemed to me that those cases in which the teeth are in direct relationship with the ear have more commonly affected the lower jaw than the upper; in fact, the three cases which I recall at this moment have all been connected with the lower jaw. The anatomical reason for this is probably that the otic ganglion, which is situated immediately below the foramen ovale, and in close relation to the structures of the middle ear, the tensor tympani muscle, and the auriculo-temporal nerve, is directly connected with the internal pterygoid branch of the inferior maxillary nerve. Exactly why pain in connection with the teeth should be referred back to the ear rather than any of the other of the points with which the inferior maxillary nerve is in relation, I cannot say. The following three cases will give evidence of this relationship.

Case I.—A Jewish woman around thirty-five years of age, came complaining of severe neuralgic pain in the right ear. Examination of the ear showed no inflammatory action whatever, and after seeing her for a couple of times I was very sure in my mind that the cause of the pain was not in the ear. I referred her to a dentist, who removed a lower molar very much out of shape and pressing directly upon the inferior dental nerve. With the removal of this tooth all symptoms referable to the ear immediately disappeared.

Case II.—A woman about the same age came complaining of severe, somewhat intermittent neuralgic pain in the ear. Examination of the ear showed no inflammatory action visible in connection with the drum, and I referred her to a dentist who removed the tooth and found the whole difficulty to be in connection with it. When I find severe neuralgic pain referred to the ear, where there are no objective symptoms suggestive of middle ear or drum inflammation, I always refer the patient to the dentist.

The third case was one somewhat different. A boy of about twelve, as a result of a cold, had an acute inflammation of the middle ear, accompanied with very severe pain. Incision of the drum was done, but there followed no relief whatever from the severe pain, which continued a number of days and required morphia hypodermically for its relief. As the boy had had a little trouble with his teeth, his dentist was called in, who found that a filling had come out of one tooth and that there was a delayed eruption of one of the bicuspids. He was very doubtful whether either one or the two together was sufficient to explain the intense pain, but after free incision of the gum and the putting in of a temporary filling in the tooth, the pain entirely disappeared, although the ear symptoms as such continued and the case later showed inflammation of the mastoid, for which a mastoid operation was done. The point here is that the severe middle ear pain which in my experience I have found to be invariably relieved by a free incision of the drum, was in no sense relieved by this, and there was no relief from the pain whatever except by the administration of morphia, until the visit of the dentist, after which the severe pain entirely disappeared. Hence it seems to me that the ear and the teeth have sufficient connection one with the other so that the aurist ought to have a certain amount of knowledge of the teeth and ability to locate in the teeth those pains for which he cannot or does not find sufficient cause in the ear; also that the dentist should recognize that certain cases of tooth trouble may produce severe ear symptoms, and be able to work intelligently with the aurist in his endeavor to locate the source of the pain.

RELATION OF MOUTH-BREATHING TO TOOTH-DEVELOPMENT.

November 21, 1895, I had the pleasure of reading before this Society a paper on the causes of mouth-breathing and the treatment to be directed for the correction of that. In the nine years which have elapsed since then, the influence of faulty nasal development and adenoid growths, not only as a cause of mouth-breathing, but as a very important factor in improper development of the upper jaw and the palatal arch, have been universally recognized, and when the dentist now looks in the mouth and finds a high, narrow arch, irregularly developed teeth, and imperfect normal occlusion, he begins to suspect that the conditions of breathing are faulty; and that there is trouble either in the nose or nasopharynx, and is quite apt to suggest that the child consult his physician or the nose and throat specialist with reference to the correction of the condition. That the adenoid growth or the imperfect nasal development is the only cause of a high arched palate or faulty normal occlusion, I would not wish to state, but that the habit of mouth-breathing dependent upon these conditions, continued during the early years of life, is an important factor in the production of this condition, is not, of course, denied by any one. When the breathing is constantly done through the mouth, the nasal passages do not develop; the floor of the nose is unduly high, and the normal nasopharynx becomes unduly small. The earlier in life the correction of this condition can be made, the better the development not only of the palate but also of the teeth.

I have in a few instances been called on to do an adenoid operation on adults of fifteen years and upward in which all the objective appearances suggested adenoids, and yet have found on examination of the nasopharynx that it was perfectly clean, contained no excess of adenoid tissue, and that the facial appearance was wholly due to faulty occlusion of the teeth, high-arched palate, and the inability to close the jaws properly, whereby the lips had a tendency to remain open. Careful teaching of these patients has usually brought about nasal respiration as there has been in each case sufficient room, and in a few instances I have found, on referring the patient to the dentist for the correction of the faulty tooth occlusion, that very much could be done for the comfort and good appearance of the patient. These cases, however, only prove the necessity of attending to such conditions at the earliest possible moment, with the presumption also that all of these patients, had they been seen in the early ages, say from four to ten, would have shown the presence of adenoid growths, as it is a well-known fact that in many cases, perhaps the majority, there is a tendency to the disappearance of these lymphoid masses around the age of puberty, though this by no means always occurs, as I have removed as large masses of adenoid tissue from full-grown, fully developed adults as I ever have from children.

The dentist therefore has it in his power, by going a little farther in his examination of the mouth than the mere attention to the teeth, to direct the individual or his parents to see to the correction of all those conditions tending to interfere with normal facial and nasal development, and the prevention of conditions which are so difficult to correct around the age of puberty and thereafter. In other words, I think it is the dentist's duty, when he has a child who is manifestly a mouth-breather, to suggest to the parents that that condition ought to be corrected, not only in the interests of the child's whole development, but in the interests of the teeth as well.

The influence of the general mouth secretions has a marked influence upon the condition of the teeth. I regard the enlarged, chronically inflamed tonsil as a menace to the well-being of the teeth and the comfort of the individual. While it is no doubt true that individuals live along very comfortably with enlarged tonsils, constantly secreting foul cheesy masses, yet the holding of these masses in the crypts tends to a foul condition of the mouth, which is often brought to the observation of the dentist when the patient himself would not seek advice therefor, and does not really recognize the condition, having become used to the discomfort. When the patient's attention is brought to this condition by the dentist, advice from him with reference to its correction will usually be welcomed. It is not always necessary that the gland itself be removed, although those of us who have been in the habit of removing tonsils for many years always find the condition of the patient better after their removal. A good deal can be done in the way of local treatment, in the cleaning of the crypts, and in the shrinkage of the tonsil by proper local measures.

It seems to me, therefore, that it lies within the province of the dentist to notice these things when he works in the mouth, and to suggest to his patient that such conditions are deleterious to the teeth and that his family physician or some specialist had better be consulted in relation thereto.

### EMPYEMATA OF THE ANTRUM.

The question of the origin of antral empyemata is one that has occasioned considerable discussion. Originally the dental profession had the entire field, but of late the nose and throat specialist is probably seeing relatively more cases as an individual than is the individual dentist, and whereas originally we thought most of them to be of dental origin, at the present time I think the consensus of opinion would rather be that the majority of them would prove of nasal rather than of dental origin, although when one comes to search the literature in regard to it, one meets with the most various opinions, varying somewhat with what has been the author's individual experience. Without wearying you with citations from a large number of authors which would have no especial value, I nevertheless wish to make a few quotations from the recent literature on the subject from three authors, and then wish to give you my own personal observations in regard to the subject.

In the *Monatsschrift für Ohrenheilkunde* of June, 1904, Dr. Alexander Strubell, of Dresden, has a very exhaustive article on the relationship of the vessels of the antrum to those of the teeth. Finding that there was the greatest variation in the opinions expressed by so-called authorities; finding also that there are apparently cases of dental origin of antral empyemata in which there is no communication with the antrum to be seen, and yet in which the clinical history would seem to admit of no doubt that such cases of empyemata had originated from the teeth, he set out to see if he could not anatomically find some relationship between the vessels of the teeth and those of the antrum, or at least some condition that is not as yet fully understood, by which the pathogenic focus could be carried to the antrum mucous membrane. There being no especial lymph-tract, only the blood-current was left, and the question then was whether there were any existing anastomoses between the blood-vessels of the alveolus and the mucous membrane of the antrum sufficiently related together to render the theory of infection from one channel to the other plausible.

In most anatomical writings there is nothing concerning this, although Zuckerkandl, in his article in Scheff's "Handbook of Diseases of the Teeth," states that the vessels that supply the alveolus communicate with those of the external periosteum; that in the upper jaw there is in addition to the external periosteum an inner one, namely, the deeper layer of the mucous membrane of the sinus, to which at the same time the arteries of the bone lead, and the veins from which lead to the bone. In a previous publication Zuckerkandl had briefly referred to something of the same sort, but as Strubell could find nothing further in anatomical and clinical literature touching this point, he made personal studies on this matter in Professor Zuckerkandl's own laboratory. Tandler's method of injection with sodium iodine, gelatin, and Berlin blue was used, and the posterior alveolar arteries were injected. The specimen was then fixed in formol, the lime salts removed in caustic potash and treated with alum, after the method of Schaffer. The sections were made from celloidin. As a result of the examination of the specimens, he found that there were three vessel systems,—a long narrow one extending to the deeper layer of the mucous membrane of the antrum, that is, its periosteal layer; a coarse-meshed vessel system of the spongy bone of the upper jaw, and a fine-meshed one corresponding to the alveolus and the covering of the tooth-root; that individually these were very characteristic, but that with one another they had such a close connection that it was very difficult to absolutely differentiate them one from the other. The junction of the vessels of the mucous membrane periosteum with that of the bone is brought about through numerous short, thick vessels coursing at short intervals in canals at right angles to the direction of the bone, while the obliquely coursing arteries of moderate size that bring about the communication between the blood system of the spongy bone with that of the alveolus are re-enforced by a large number of the finest blood-vessels. Here it is seen that the fine capillary system of the spongy bone, whose vessels lie in the marrow of the bone, are connected with those of the alveolus, and prove substantially the theory that the three systems are in foundation one and the same. Hence it seems proper to say that the nourishment of the antrum and its periosteum, the spongy portion of the upper jaw, and the teeth in the alveolus all come from one set of vessels which anastomose with one another in the most extensive way. Hence it is easy to see that inflammation may travel from the antrum to the teeth or from the teeth to the antrum without there being a direct continuity of surface or a direct infection from one diseased area to the other.

Strubell then goes on to discuss the question of the relative preponderance of origin between dental and nasal causes, giving the statistics which follow, all of which from European authorities I take from his article with the exception of those of Tilley and Lermoyez, the source of which is stated.

Bayer, of Brussels, thought the most frequent cause of empyema to be disease of the teeth.

Grünwald considered twenty-one out of thirty cases to be dental in origin.

Schiffers, Goodwillie, Moreau, Schmidt, Baginsky, Lublinski, M. Schmidt, Krieg, Schütz, Christopher Heath, Scheff, Heryng, and Schmiegelow, as the result of their clinical observations, consider the teeth to be an etiological factor.

In an article read before the Odontological Society of London, November 23, 1903 (*Laryngoscope,* February, 1904), by Herbert Tilley, a prominent London laryngologist and surgeon to the Golden Square Hospital for the nose, throat, and ear, the one founded by the late Sir Morell Mackenzie, Dr. Tilley reports briefly on sixty-four cases of antral suppuration seen in private practice, and eighteen hospital cases seen since January 1, 1902. Of the sixty-four cases seen in private practice, upon which his paper is based, he found diseased bicuspid or molar teeth to be present, or by their absence implying previous removal for disease, in every case, with one exception, upon the side corresponding to the antral suppuration. He says, further, that during the past ten years, during which he must have seen at least three hundred cases of antral abscess, he has only met with one patient, a girl aged twelve, in whom the teeth were quite healthy.

B. Fränkel, as a result of his clinical experience states that in by far the larger number of cases disease of the teeth and alveolæ were the causative factor. For his position he states that he had never seen a case of empyema of the antrum in which the corresponding tooth had not been previously extracted or was at least diseased. This would agree with the observation of Herbert Tilley just quoted. And his final conclusion is that empyema of the antrum usually has its origin through the alveolus of the teeth.

Hartmann, in one article, stated that in sixteen empyemata only one case originated from a carious tooth. He reported one case of empyema of the antrum where, as the result of a filling, or following the filling of a second upper molar, pus developed. The tooth was extracted. It was then easy with a few turns of the borer to go directly from the alveolus into the antrum, after which quite a quantity of foul pus was removed. Under daily washing, healing followed a few weeks after. In another article Hartmann stated that in most cases carious teeth formed the etiological moment for the antral empyema, but that it was not always possible to determine whether the inflammation of the tooth was the real cause of the inflammation of the antrum. In only two cases after extraction of the tooth did he find an opening directly into the antrum. The fact that, in spite of a previous inflammation of the roots of the tooth, healing of an empyema of the antrum has been brought about through washing out of the nasal cavity, allows us in many cases to say that there is probably no direct union of the antral cavity with the carious process. In one-third of Hartmann's cases there were polyps in the nose, and in these he thought there was a nasal origin of the affection. In twenty-three out of thirty-two cases healing was brought about through regular washing out through the middle fossa of the nose.

Lermoyez (*Journ. Laryngology,* 1902, p. 576) states that dental origin is more frequent than nasal origin. This is due to the sudden rupture of the radiculo-dental periapexial abscess into the cavity of the sinus. It is only subsequently that the empyema is transformed into a chronic sinusitis.

Those authors who think that the inflammation comes from the teeth base their propositions on the fact that many tooth-roots, especially those of the first and second molar, reach almost to the periosteum of the antrum and are separated from it by a very thin plate of bone, while in others even this thin plate is not present and the roots lie in direct contact with the soft parts of the antrum. Caries of these teeth, therefore, leads easily to the periosteum, and not seldom to the formation of pus. Inflammation, therefore, can go directly to the mucous membrane of the antrum. Inasmuch, however, as almost all people have carious teeth, and as empyema of the antrum is comparatively seldom, this argument rather loses its force. It is perfectly possible that a carious condition can be present before the toothache appears. Even if after the extraction of a tooth an empyema appears evident that previously has given no particular signs of its presence, nevertheless it is perfectly possible that that empyema could have lasted several years. The communication between the teeth and the antrum can have grown up, and this be reopened by the extraction of the tooth. I think that in many cases of chronic empyema this is probably just what happens.

Coming next to the other theory,—namely, that the inflammation can pass from the nose directly to the antrum,—I think that the majority of present-day authors would rather hold to the preponderance of the nasal origin of antrum trouble.

The anatomist Zuckerkandl considered that antral empyemata of dental origin occurred very seldom. In his anatomy of the nose he speaks of the importance of the dehiscence of the infraorbital canal in the antrum, and states that in such cases the entire tract of the tooth nerves is in direct relationship with the antrum covering, and in case of disease of the sinus the mucous membrane can be pressed upon or infected. When this occurs, there would, of course, be pain, first referred to the tooth and supposed to be a toothache, especially if there should happen to be on that side a bad tooth. As a proof of his belief that the inflammation could pass directly (especially purulent inflammation) from the nose to the antrum, the following facts are cited.

First, the presence of cases of empyema that followed sound teeth.

Second, purulent inflammations of the sphenoid and frontal sinus.

Third, the presence of primary and inflammatory inflammation of the nasal mucous membrane.

Dmochowski examined three hundred and four maxillary sinuses without seeing a single case of empyema of dental origin, although he always chiselled away the maxillary process.

In thirty-nine upper jaws examined by Weichselbaum he found the first bicuspid carious in six cases, absent in eight cases, sound in twenty-five cases; second bicuspid, carious in ten cases, absent in seven cases, sound in twenty-two cases; first molar, carious in fourteen cases, absent in eleven cases, sound in fourteen cases; second molar, carious in nine cases, absent in nine cases, sound in twenty-one cases.

Krause considered that carious teeth did not bring about empyema.

Jeanty, in twenty-one cases where there were carious teeth, in connection with twenty-two cases of empyema, considered that in no case were the teeth the causative factor.

Moreau, in twenty-one cases of empyema, found only eight connected with caries of the teeth.

Killian reports forty cases of inflammation of the antrum,—fifteen men, twenty-one women; with forty-four antral empyema,—eighteen right, twenty-six left. Concerning the dental origin, the history of many was in doubt. Several thought the disease to have been due to ulceration of the teeth or brought about by tooth extraction. In several there was not the slightest complaint of the teeth. In four acute cases three had toothache; one with entirely sound teeth. Killian thinks the only way to get solution of the problem is not through the varying clinical observations, but through direct pathological and anatomical examinations to find a direct way in which an inflammation can travel from the teeth to the mucous membrane of the antrum, by examination of properly prepared specimens as well as through bacteriological work.

In four of Killian's patients, after extraction of the teeth there was direct communication with the antrum. In two there was only a very thin mucous membrane and a very thin bony layer between the tooth and the antrum. In this case it was the second bicuspid. In four it was the first molar, and in another the second molar.

As the observations seem to render probable the infection of the antrum from diseased teeth, one has also to think, in the case of inflammation from the teeth (especially in cases where the bottom layer or even the mucous membrane of the antrum touched the tooth), whether, as a matter of fact, an empyema must be produced. Killian doubts this, for an abscess at the root of the tooth can last a long time without breaking through into the antrum. In such a case the mucous membrane itself can be pushed far forward, and it is also possible to imagine that the tooth can be extracted and the abscess wall opened without breaking through into the antrum. In the case of granulations or fungosity of the roots, as in the case seen by Killian, where the second bicuspid was carious with a hazel-nut granulation on the root, this opened directly into the antrum, and after its extraction there was a correspondingly large communication between the alveolus and the antrum; nevertheless the washing out of the antrum gave no sign of any pus.

Finally, one must also consider the possibility that an inflammation of the nasal mucous membrane itself can be brought about as a result of the antrum empyema, the same way as by influenza. If the nose is absolutely sound; if influenza and the like can be ruled out, and where there is nothing to be considered except the diseased tooth, then it is more than probable from the clinical stand-point that the tooth was the cause.

Hajek, one of the principal workers at the present time in rhinology, thinks the presence of an alveolar sinus with a very thin lamella of bone over the tooth to be the principal cause of the origin of dental empyema. The infection of the antrum can be brought about by abscess of the tooth, inflammation of the pulp as the result of caries or from filling, purulent inflammation of the periosteum of the alveolar process, and cysts of the root with purulent contents. Oftentimes the empyema manifests itself through the extraction of a tooth because at the same time the antrum is opened and allows the pus to come out. In only thirteen cases out of approximately two hundred antrum cases that Hajek has seen in the last eight years, does he regard the origin of the same to have been due to dental causes.

Zuckerkandl and Dmochowski also advance the proposition that it is perfectly possible that many cases of empyema may be not the result of the tooth affection, but, on the contrary, that the tooth affection may be the result of carious and pathological processes in the antrum itself, since the nerves and vessels of the antrum which supply a portion of the teeth have direct relations with the soft parts of the maxillary sinus, the deeper parts of the bone.

I have quoted thus somewhat freely from Strubell's article, interspersing it with comments of my own, because the subject is one of considerable importance, and I think these quotations show that any definite statement in the mass as to whether empyemata are of dental or nasal origin is positively worthless. Each individual case must be worked out for itself, and it is in the working out of the individual case that I think the co-operation of the rhinologist and the dental surgeon is required. The dentist must not lose sight of the fact that many cases of empyema are of extra-dental origin; the rhinologist must not lose sight of the fact that not all cases of empyema are of nasal origin.

Coming from foreign to American authors, I think it will be found, on careful scanning of the latest literature, that the prevailing opinion among rhinologists is coming to be that the majority of cases are of nasal origin. In Cryer's book on the "Anatomy of the Face," he states, page 73: "This close proximity between the apical portions of the roots of the teeth and the sinus gives the impression that the maxillary sinus is oftener infected from diseased teeth than from any other source, some authorities claiming that three-fifths of the diseases of the antrum are brought about in that way. The writer thinks this is a mistake. Though recognizing that diseases of the antrum do arise from the teeth, he believes that, aside from constitutional diseases and malformations, it is more often through the common communication between the nasal chamber, the frontal sinuses, the ethmoidal cells, and the maxillary sinus that infection is conveyed to the antrum from diseased cells and sinuses above it. It is the writer's observation that there are more cases in which teeth are lost through diseases of the antrum than cases in which the teeth are primarily diseased, causing infection of the antrum and associated cells.

"Pus or infected matter will, of course, pass in the direction of the least resistance. When the investing tissues of a tooth become so infected, the osteogenetic layer of the muco-periosteum stimulates renewed activity, with the result that a new layer of bone is produced by it which covers these parts and protects this cavity so that abscesses, with but few exceptions, point and break into the mouth.

"Careless operation by the dentist sometimes causes infection of the sinus, as drilling through the tooth and the floor of the sinus, or forcing the root of a tooth into the sinus, through fracture of the wall in an unskilful effort to extract, or carelessness in driving artificial crowns or bridges upon the teeth or roots."

I have tabulated thirty cases of empyema of the antrum seen by me in private practice in the last eight years, with the following results: Sixteen of them were chronic cases of undoubted nasal origin in connection with general ethmoiditis, with inflammation of the frontal sinus, or with atrophic rhinitis. One of these had syphilis, but the syphilis seemed to be a coincident condition rather than in any way the cause of the general ethmoiditis. Three were the result of influenza of nasal origin. One was traumatic, the result of a railroad injury. One was luetic, the syphilis causing a breaking down of the naso-antral wall on one side and a great thinning of it on the other. Six were of undoubted dental origin, while three were possibly of dental origin. Or, reduced to percentages, 63.3 per cent. were undoubtedly of nasal origin; thirty per cent. of possible dental origin; 3.3 per cent. traumatic; 3.3 per cent. luetic.

It is extremely difficult in many cases where the empyema is a chronic condition and diseased teeth and general ethmoiditis are both present to determine which was the primary cause and which was a coincident or subsequent condition. Since carious teeth are very common in persons who do not have and never have had any nasal trouble, it is quite possible that the two processes could work along independently of one another. I think this probably occurred in one of the cases which I have stated to be of dental origin, and that really the nasal condition was the primary cause. In one of the cases which I have stated as distinctly of dental origin, this might reasonably be doubted, as no distinct trouble could be found with the tooth on its removal, and the dentist who removed it hardly considered that there was anything the matter with it. At the same time there was no general ethmoiditis, and the removal of the tooth relieved the condition of pain which was present in the antrum. On the whole, I think that in my analysis I have allowed quite as many of my cases to be of dental origin as the facts would warrant. In a recent article ("Pus in the Nose," *Medical News*, December 24, 1904) I stated that I thought my experience had shown that approximately fifty per cent. of my cases were of dental origin, but a careful scrutiny of them shows this percentage to have been altogether too high. From twenty-five to thirty per cent. is probably much nearer the correct figure.

There are many cases of acute inflammation of the antrum of influenzal origin which are seen by every rhinologist, but of which no account is made here. In the three acute cases which I have tabulated pus was actually washed out of the antrum. The acute cases in which there were antral pain and all the symptoms of acute non-purulent inflammation of the antrum I have disregarded.

So much, then, as to the origin of these affections. We come next to an equally important section of the subject: What shall be the treatment of the empyema of the antrum when the diagnosis is made? If we look up the history of cases reported even as late as five or six years ago, we will find that, preceding the days of more radical nasal surgery, the majority of these cases were all treated through openings through the alveolar process made by the extraction of the tooth, with an enlargement of the opening and its maintenance by means of a tube. In process of time a good many of them got well. Others, however, were condemned to wear the tube almost indefinitely. Tilley reports only five cases out of twenty-two that could give up the tube after having used it a long time. Of twenty-seven alveolar cases, fifteen had worn the tube with careful irrigation for at least six months. One patient had worn it ten years; another three and one-half years, and another two years.

I think we have now come to the point where this method of treatment in cases which have lasted any length of time must be considered as distinctly obsolete. Any one who has done a number of radical operations on the antrum, and has seen the character of the degenerated mucous membrane which is found there, often the entire antrum being filled with numerous polyps and various low formations of granulation tissue, will thoroughly appreciate the uselessness of expecting to get any cure through a small tube opening into the mouth, even though that tube may satisfactorily drain the pus. Again, why should a patient be compelled to syringe out his antrum, day in, day out, when a radical operation, the healing of which will be complete in a few weeks, can be performed and the case be disposed of once for all?

Without discussing further this subject of treatment as to its pros and cons, I will tell you the method which I am in the habit of pursuing and which I believe, in the light of our present knowledge, is the proper one. Given a case of supposed antral empyema, first, as to diagnosis. The presence or absence of pus can be determined pretty quickly and very easily by puncturing underneath the inferior turbinate as high up as possible and at the junction of its anterior and middle thirds, although there will be a few cases in which the antrum may be so filled with polypoid growth, with comparatively small secretion, that the diagnosis here may for a short time be somewhat in doubt. In most instances, however, the diagnosis is definite enough.

In the second place, have the case examined carefully by a dentist to see if there is any trouble with the teeth. If the tooth is at fault, extract it. See whether it opens into the antrum. Assuming that it does, and that the tooth is the causative factor, is the antrum acutely or chronically affected? If acutely affected, it will get well in a short time with daily washings out with the usual antiseptic fluids. If it lasts more than a reasonable time, two or three weeks, or even less, without manifest cessation, I regard the case as chronic, and further treatment by way of the teeth as useless. At the same time that the dentist was treating the teeth, if the patient was under my own observaion, I should have enlarged the original exploratory opening made in the nasal wall of the antrum, by means of curettes and alligator cutting forceps, so as to get a free opening from the antrum into the nose, thus securing free drainage, which drainage is very much superior to that through the mouth. The short curettes, bent slightly on the shank, which are used for the radical mastoid operation and are of various sizes, and the smallest and second size Myles's aligator cutting forceps answer very well for this purpose. If the case lasted any considerable length of time, I should next do a radical operation, taking off, under ether, sufficient of the front wall of the canine fossa, so that free inspection of the antrum cavity could be had; its entire contents, including the mucous membrane, removed if necessary, and a large counter-opening then made directly into the nose underneath the anterior end of the inferior turbinate; the natural opening also enlarged, and the whole antrum cavity packed with gauze. The canine fossa opening is allowed to close in a few days, thus sealing off the cavity of the mouth, and the nasal opening is retained as the permanent one. The details of this operation I have described in my book on the nose and throat[1] and in the article in the *Medical News* already referred to.

This operation, of course, lies in the province of the rhinologist rather than of the dentist, but the entire working out of the pathol-

[1] "Nose and Throat Work for the General Practitioner," New York, 1903.

ogy and treatment in the borderland cases should be made by the dentist and the rhinologists together.

There is another class of antrum cases which might have been considered in our discussion of the etiology,—namely, those in which the antrum is infected from the frontal sinus by reason of the drainage of the pus from frontal sinus empyemata, down through the infundibulum, and then into the natural opening of the antrum, or directly into the antrum, a condition which occurs, and so frequently that I now believe that all cases of radical operation of the frontal sinus should have at the time of operation, or previously, exploratory puncture of the antrum to determine whether or not the antrum is at the same time affected.

The after-treatment of the cases in which a radical operation is done is comparatively simple, does not last a great while, and has in my hands been invariably successful.

### SURGERY OF THE ALVEOLAR PROCESS.

The rhinologist comes in contact with the dentist in another division of his work,—namely, in alveolar abscesses, the extraction of teeth, and affections of the jaw. In quite a number of instances in the last few years patients have come to me complaining of pus underneath the mucous membrane of the upper jaw, in which the history seemed to show a certain degree of infection of the parts at the hands of the dental surgeon. In nearly all of these instances it seemed to be due to the laudable endeavor of the dental surgeon to save teeth which, from the stand-point of the rhinologist, had better have been extracted. I am quite aware, so far as this phase of the subject is concerned, that there may be great difference of opinion, and I will probably be told that the dental surgeon saves a great many teeth which the rhinologist would have pulled, and that in only a few instances is there any after-trouble.

I have recently had as a patient a boy of twelve or fourteen. He had had a tooth extracted some eight months before, a first left upper molar, and later developed a small abscess of the cheek. He was seen by two other dentists, neither of whom seemed to be able to cure the condition. He was referred to me by a general surgeon, into whose hands he fell, as a probable case of antrum trouble. At the time he came he had a granular ulcer the size of a ten cent piece disfiguring his cheek, at the bottom of which ulcer cavity some bare bone could be felt, but which did not penetrate the antrum.

There was nothing in the contour of the upper jaw to suggest that there was any tooth-root present. A competent dental friend of my acquaintance, to whom I took the case and to whom I suggested that there might be a tooth-root there, doubted its presence. I took the boy to the hospital, etherized, curetted, and extracted a carious tooth-root with an abscess at its tip. The further history of the case is uneventful. Suffice it to say that we fortunately got healing with comparatively little scar.

Another instance. A patient came with severe pain in the upper jaw, with all the signs of an alveolar abscess, but with no pus evident and no swelling. I punctured through the alveolus, found pus bathing the tooth-root, extracted the tooth, and found that the dental surgeon, in getting ready to cap this tooth, had drilled through it instead of up to the apex of the root, and had infected the surrounding tissue with the result as stated. He had seen the case two or three times before it came to me, but had neglected to do anything. I would like to make the criticism here that, as I see them, the average dentist is not sufficiently courageous in his surgery of the region around the teeth; that when he opens an abscess he does not make a sufficiently large opening; that when he drills through the alveolar process to a tooth-root in the endeavor to save that tooth, his opening is too small; that his entire surgery, the moment he is away from the tooth, is too timid. The larger the opening, the less the pain, and as in modern days we have to anæsthetize more or less anyway, if we do anything that is likely to produce pain, it means but little more primary pain to the patient to make a good opening, whereas afterwards the comfort is much greater. These last two cases are only examples of quite a number which have come under my observation.

SYPHILIS.

The last topic which I think is of common interest relates to the subject of syphilis. I wonder how many of my dental friends feel competent to diagnose the mouth lesions of syphilis; and if there are any here who do not, I would suggest that they take a course in it at some one of the dispensaries, or study up the subject with some physician of their acquaintance who is versed in the subject.

We have come to pay more and more attention to the care of our instruments, apparatus, etc., and I presume there is no dentist present who would endanger his patients in any way by the using of one set of instruments on another without their thorough sterilization. But beyond that there is the danger of the dentist's infecting himself from the syphilitic mouth and from the syphilitic saliva. In working on the mouth it is very easy to get a slight abrasion in one's own fingers or the fingers of one's assistant, and the syphilitic virus is quite as easily transmitted by way of the mouth tract as by the more commonly supposed one of the sexual act. I think that every dentist owes it to himself and his patients to recognize on the first inspection the commoner syphilitic lesions of the mouth. Within a fortnight a patient has come to me with a sore throat showing most beautifully all the secondary lesions of syphilis, its most contagious stage, with mucous patches on both tonsils, and as I finished with him he said, "Well, I am going to the dentist's now to have a tooth pulled." I inquired what dentist he was going to, but he didn't know. Had it been one of my own friends I think I should have sent a special messenger to him to take extraordinary care. Within a few years I have had another well-marked case of syphilis tell me that she got it through unclean instruments used by the dentist in the extraction of a tooth. More careful inquiry, however, revealed the source had been somewhat otherwise.

That this problem of the syphilitic virus in the mouth probably is of more moment than is generally considered, and that the dentist more often operates on the syphilitic mouth than he thinks, may be proved by the fact that in a single morning in my own private practice I have seen five cases of syphilis of the upper air-tract, and that in an ordinary private practice not exceptionally large I have always under my care from one to three or four such cases. In my own practice my assistant is always informed of the nature of the case, so that she may take extra precautions in reference to instruments, linen, and everything which touches such a case. No linen or towel is ever used on those cases except such as is thrown away, and the table and everything in connection is especially prepared.

The time at my disposal does not allow me to go into the details as to the diagnosis of these conditions. That can be obtained easily in the treatises on syphilis and from the text-books on the nose and throat. I would urge, however, upon every dentist that he make himself perfectly familiar with the commoner lesions of secondary and tertiary syphilis, and this is not difficult.

REPORT OF THIRTY CASES OF EMPYEMA OF THE ANTRUM.

| Name. | Age. | Sinus involved. | Operation. | Date first seen. | Date last seen. | Result. | Remarks. | Origin. |
|---|---|---|---|---|---|---|---|---|
| Mr. F. W. N. | 32 | R. frontal. R. antrum. | Right frontal operated on by external incision. Antrum washed out. | Nov. 4, 1898. | Jan. 7, 1901. | Cured so far as known. | Antrum was originally free from pus; became infected from frontal. | Nasal. |
| Miss M. B. | 25 | R. antrum. | Exploratory puncture underneath inferior turbinate. Daily washings. | Sept. 25, 1896. | May 11, 1897. | Cured so far as known. | Had atrophic rhinitis also. | Nasal. |
| Mr. D. H. C. | 66 | R. antrum. | Exploratory puncture under inferior turbinate. Silver canula. Daily washing. | Oct. 3, 1896. | Mar. 14, 1898. | Cured so far as known. | Is in apparently good health. | Nasal. |
| Mr. W. H. | 32 | R. antrum. | November 4, 1902, puncture of antrum showed pus. Daily washing without effect. December 15, 1902, radical operation through canine fossa with extraction of first molar tooth. | April 18, 1898. Complained of catarrh. | May 28, 1903. | Cured. | First molar tooth penetrated floor of antrum and had a pus pocket attached. Antrum walls not specially diseased. | Dental. |
| Mrs. S. | 64 | L. antrum. | Exploratory puncture; daily washing for two months. Improved, but no cure. Then removed second molar, and made large incision through the alveolar process. Cure in a few days. | Feb. 5, 1897. | May 3, 1897. | Cured. | Complained at no time of tooth which was filled with amalgam. | Dental. |

| | | | | | | | |
|---|---|---|---|---|---|---|---|
| Miss J. S. | 24 | R. antrum. | Puncture underneath the inferior turbinate and washed out cavity. | June 17, 1899. | Dec. 9, 1899. | Slight discharge at time case lost sight of. | No trouble with teeth. Antrum very small. | Nasal. |
| Mr. G. D. F. | 26 | R. antrum and R. frontal. | Washed out antrum to no purpose; then did radical antrum and radical frontal. | June 21, 1899. | April 6, 1904. | Cured. | Case traumatic in origin from railroad accident. | Traumatic. |
| Mrs. M. G. | 37 | Both frontals. Both antra. | Various operations on frontal sinuses; antra secondarily infected from these. | Oct. 17, 1899. | July 12, 1904. | Cured. | Extensive ethmoiditis several years duration. | Nasal. |
| Mrs. F. | 41 | L. antrum. | Radical from antrum through canine fossa, syringing having failed to effect cure. | Dec. 13, 1899. Then occasionally until Apr. 11, 1904, when radical operation was done. | Oct. 28, 1904. | Cured. | Originally thought was case atrophic rhinitis simply. Pain from pressure on infraorbital nerve a prominent after-symptom. Disappeared in three weeks. | Nasal. |
| Mr. M. S. | 24 | L. antrum. L. frontal. | March 7, 1900, radical through canine fossa. Antrum filled with soft, pulpy granulation tissue. Second bicuspid absent. | Mar. 2, 1900. | Sept. 1904. Antrum clear. | Cured as to antrum by first operation. | Frontal was afterwards operated on, and again three years later. Permanent cure now effected. | Nasal. |
| Mrs. N. A. | 25 | R. antrum. | Radical through canine fossa and also through alveolar process at site first bicuspid previously removed. | June 9, 1900. | Aug. 4, 1900. | Cured. | First and second bicuspids and first molar absent at time of operation. | Nasal or dental (probably dental). |

| Name. | Age. | Sinus involved. | Operation. | Date first seen. | Date last seen. | Result. | Remarks. | Origin. |
|---|---|---|---|---|---|---|---|---|
| Mrs. A. C. | 39 | L. antrum. | Radical through canine fossa and also through alveolar process at site first bicuspid previovsly removed. | June 12, 1900. | Nov. 4, 1902. | Cured. | Wore gold tube through alveolar opening for awhile as no opening was made into nose. It would have been better to have made one. | Dental. |
| Miss M. D. | 29 | L. antrum. | Exploratory puncture. | April 5, 1902. | Under treatment for atrophic rhinitis, but antrum has no pus. | | | Nasal. |
| Miss A. A. | 24 | L. antrum. | Exploratory puncture. | Oct. 23, 1902. | April 16, 1903. | Cured. | | Nasal. |
| Miss H. D. | 26 | Both antra. Had general ethmoiditis with polyp formation. | Radical both antra. April, 1904. | Nov. 21, 1902. | Sept. 3, 1904. | Cured. | At time of operation three carious roots were found penetrating left antrum. As both antra were diseased and no trouble with teeth on right side, doubtful if tooth was cause. | Probably nasal, perhaps dental. |
| Miss O. M. | 17 | R. antrum. R. frontal. | Radical operation frontal sinus and antrum. | Feb. 11, 1902. | June 9, 1903. | Cured. | Operated on frontal first, then did radical antrum as pus discharge did not stop. Think frontal infected antrum. | Nasal. |

| | | | | | | | | |
|---|---|---|---|---|---|---|---|---|
| Mr. W. G. | 29 | R. antrum. R. frontal. | Radical for each. | Mar. 22, 1903. | Dec. 14, 1904. | Apparently cured. | Impossible to say whether frontal or antrum primary source of trouble. Had general ethmoiditis. | Nasal. |
| Dr. G. L. R. | 40 | L. antrum. | Exploratory puncture. Daily washing. | Jan. 1903. | Three weeks duration. | Cured | Absolute cure in three to four weeks. | Nasal (influenza). |
| Mr. A. J. F. | 25 | R. antrum | Exploratory puncture. | Mar. 10, 1903. | Mar. 12, 1903. | Cured. | Acute case influenzal origin. | Nasal. |
| Mr. E. P. | 21 | Both antra. | Exploratory puncture L. Right side naso-antral wall already perforated. | June 27, 1903. | Nov. 7, 1903. | Improved. | Result tertiary lues. | Luetic. |
| Mr. L. W. J. | 24 | Both antra. Both frontals. | Radical operation all four cavities June 17, 1903. All sinuses filled with polypi and degenerated mucous membrane. | | Dec. 1904. | Cured apparently. | No tooth complications. | Nasal. |
| Mr. M. W. M. | 25 | L. antrum. L. frontal. | Exploratory puncture antrum showed pus. Frontal sinus also involved. | Oct. 24, 1903. | Nov. 24, 1903. | Relieved. | Middle turbinate was removed. This improved drainage. No other treatment allowed. | Nasal. |
| Mr. H. S. H. | 35 | R. antrum. | Radical operation R. antrum. | Feb. 10, 1903. | Dec. 22, 1903. | Cured. | Luetic with general ethmoiditis and polypi each nostril. No tooth symptoms. | Nasal. Luetic. |
| Miss E. A. | 49 | L. antrum. | Removed portion middle turbinate followed by radical antrum operation. | April 21, 1903. | July 14, 1903. | Cured. | Had first molar tooth pulled but root found to be healthy and not penetrating antrum. | Nasal. |

| Name. | Age. | Sinus Involved. | Operation. | Date first seen. | Date last seen. | Result. | Remarks. | Origin. |
|---|---|---|---|---|---|---|---|---|
| Mrs. W. W. | 68 | R. antrum. | Exploratory puncture and daily washings; removal middle turbinate. | Nov. 19, 1903. | Dec. 12, 1903. | Improved. | Had many nasal polypi and extensive ethmoiditis. On account of very poor general health no further operation allowed. | Nasal. |
| Miss M. T. | 32 | L. antrum. | Exploratory puncture and daily washing out with canula. | Mar. 5. 1904. | Mar. 19, 1904. | Cured. | Acute case from influenza. No return eight months later. | Nasal. |
| Miss F. H. | 36 | R. antrum. | Second bicuspid extracted; showed old inflammation at root but did not penetrate the antrum. Then drilled into antrum. This opening closed in two days. The exploratory opening from the nose then enlarged with curettes and cutting forceps. | May 24, 1904. | Sept. 3, 1904. | Cured. | This may have been primarily of dental origin as pus had lasted some time. After establishing free nasal drainage cure was rapid. | Nasal or dental. |
| Mrs. N. B. B. | 50 | R. antrum. | Removed first molar; showed root abscess with opening directly into the antrum. | April 18, 1904. | May 20, 1904. | Cured. | After treatment through root cavity only. | Dental. |

| | | | | | | | | |
|---|---|---|---|---|---|---|---|---|
| Mrs. D. V. C. | 30 | R. antrum. | Exploratory puncture through nose. Second bicuspid, a capped tooth, then removed. No abscess, but penetrated antrum. | April 2, 1904. | April 15, 1904. | Cured. | Influenza closed natural opening. Pus probably of dental origin, but gave no sign. Many patients do not notice slight purulent discharge, considering it a symptom of what they call catarrh. | Dental. |
| Mr. W. E. Y. | 37 | R. antrum. | Exploratory puncture under inferior turbinate. Removed second bicuspid. No apparent connection with antrum but relieved pain. Cut away portion naso-antral wall with cutting forceps. | April 23, 1904. | Sept. 10, 1904. | Cured. | Had complained of catarrh for some time with some discharge. | Possible dental. No ethmoidal trouble. |

Note.—Since tabulating the above a case of double empyema of the antrum in a young woman of twenty-three has come under observation. All of the teeth in the upper jaw were sound. The antral empyema seemed secondary to a general ethmoiditis with polyp formation. A portion of the naso-antral wall was cut away under the inferior turbinate, and treatment carried out through the nose.

